# Middle Cherokee Medical Association

**Published:** 1889-09

**Authors:** 


MEETING FOR THE ADJUSTMENT OF FEES.—An important move was made among the medical men of Adairsville, Ga., and surrounding territory, on the 10th, The meeting was called for the purpose of establishing a universal fee bill and to perfect a permanent organization of the practitioners of the Middle Cherokee Section, for the upholding and advancement of the faculty. It was a meeting of enthusiasm and work. The association will embrace all the section from the Oostanaula River to the Etowah. At our first meeting, Cartersville, Cassville, Kingston, Folsom, Nannin and other places were represented. Doctor John W. Bowdoin was elected President; Joe P. Bowdoin, Secretary and Treasurer. Drs. Jos. W. Bradley, Battle, Jones, Griffin and King were appointed committee on fees. Drs. Jones, Battle, J. D. Bradley, Bufford and J. W. Bradley, on laws, constitution, etc. It is probable that the constitution of the Atlanta Society will be adopted. With such men in the lead it is useless to say it cannot but be a success. The next meeting will be held on Saturday, September 21st, 1889, at Kingston. A full attendance is expected and many new members will be received. The influence of this association will be felt throughout Georgia, for it will fill a place that has been void. It makes each man's fees the same, thereby divesting the profession of the objectionable feature of traffic. Why can't the servants of the people, we poor country doctors, succeed in business ? Why can't we gain enough of this world's goods that at our departure from this mundane sphere, our wives and children will have a reward. Few there be that do so, yet we can by unity of action, and we expect to at Adairsville. If the profession throughout the State will unite on the plan we have adopted, backbiting will cease and brotherly love increase. Universal fees and they at a figure all can live by, and which are satisfactory to the patients at large, will accomplish wonders. Keep your eye on the Middle Cherokee Medical Association.

